# Serum MG53/TRIM72 Is Associated With the Presence and Severity of Coronary Artery Disease and Acute Myocardial Infarction

**DOI:** 10.3389/fphys.2020.617845

**Published:** 2020-12-17

**Authors:** Hongyang Xie, Yaqiong Wang, Tianqi Zhu, Shuo Feng, Zijun Yan, Zhengbin Zhu, Jingwei Ni, Jun Ni, Run Du, Jinzhou Zhu, Fenghua Ding, Shengjun Liu, Hui Han, Hang Zhang, Jiaxin Zhao, Ruiyan Zhang, Weiwei Quan, Xiaoxiang Yan

**Affiliations:** ^1^Department of Vascular and Cardiology, Ruijin Hospital, Shanghai Jiao Tong University School of Medicine, Shanghai, China; ^2^Institute of Cardiovascular Diseases, Shanghai Jiao Tong University School of Medicine, Shanghai, China; ^3^Department of Nephrology, Zhongshan Hospital, Fudan University, Shanghai, China

**Keywords:** MG53, coronary artery disease, acute myocardial infarction, biomarker, risk stratification

## Abstract

**Background:** Mitsugumin 53 or Tripartite motif 72 (MG53/TRIM72), a myokine/cardiokine belonging to the tripartite motif family, can protect the heart from ischemic injury and regulate lipid metabolism in rodents. However, its biological function in humans remains unclear. This study sought to investigate the relationship between circulating MG53 levels and coronary artery disease (CAD).

**Methods:** The concentration of MG53 was measured by enzyme-linked immunosorbent assay (ELISA) in serum samples from 639 patients who underwent angiography, including 205 controls, 222 patients with stable CAD, and 212 patients with acute myocardial infarction (AMI). Logistic and linear regression analyses were used to analyze the relationship between MG53 and CAD.

**Results:** MG53 levels were increased in patients with stable CAD and were highest in patients with AMI. Additionally, patients with comorbidities, such as chronic kidney disease (CKD) and diabetes also had a higher concentration of MG53. We found that MG53 is a significant diagnostic marker of CAD and AMI, as analyzed by logistic regression models. Multivariate linear regression models revealed that serum MG53 was significantly corelated positively with SYNTAX scores. Global Registry of Acute Coronary Events (GRACE) scores also correlated with serum MG53 levels, indicating that MG53 levels were associated with the severity of CAD and AMI after adjusting for multiple risk factors and clinical biomarkers.

**Conclusion:** MG53 is a valuable diagnostic marker whose serum levels correlate with the presence and severity of stable CAD and AMI, and may represent a novel biomarker for diagnosing CAD and indicating the severity of CAD.

## Introduction

Cardiovascular disease (CVD) is one of the most common causes of mortality and morbidity worldwide (Lu et al., 2012). It has been estimated that more than 23.3 million people will die every year from cardiovascular diseases by the year 2030 (Mathers and Loncar, 2006). Coronary artery disease (CAD), and one of its most severe complications, acute myocardial infarction (AMI), are major subsets of CVD. Stable CAD (sCAD) and AMI have the same pathological background, although there are conspicuous differences in their pathogenesis and symptoms (Agewall, 2008; Libby, 2013). Atherosclerosis is a potential cause of CAD. Stable CAD refers to patients with progressive atherosclerotic plaques in coronary vessels whose clinical symptoms are stable on treatment. The diagnosis of stable CAD is generally based on symptoms or invasively angiography findings. However, these examinations are affected by the influence and bias of the performer, and are associated with some complications (Newby et al., 2012). Assay of cardiac biomarkers using conventional diagnostic methods to predict CAD events does not meet the requirements of sensitivity and specificity (Morse, 2015).

Stable plaques can evolve into unstable vulnerable plaques, which are prone to rupture and thrombosis, and may finally lead to acute myocardial infarction in conditions, such as ischemic cardiomyopathy. AMI can cause cardiac rupture, lethal arrhythmia, and heart failure (Negre-Salvayre et al., 2017). Although AMI can be successfully diagnosed using biomarkers, such as cardiac troponin and creatine kinase (CK), the usefulness of some of these conventional biomarkers in the acute stage of AMI are limited as they could have low concentrations at this stage (Suzuki et al., 2018). Hence, there is a persistent demand for biomarkers that can identify patients with AMI in the acute phase. Furthermore, the lack of protein-based biomarkers that can be used for risk stratification, is also an unmet need that could have increased the ability of biomarkers to identify patients with higher risk of adverse outcomes.

Mitsugumin 53 (MG53, also known as TRIM72) is a tripartite motif protein that was first identified in an immuno-proteomic library aimed at discovering proteins that correlated with myogenesis, ion channel, and muscle integrity (Rudnicki et al., 2008; Weisleder et al., 2008). It belongs to the TRIM family, which plays a key role in a large range of biochemical activities such as cell proliferation, differentiation, and immune response (Ozato et al., 2008; Doyle et al., 2010; Hatakeyama, 2011). The biological function of MG53 was first reported by Cai et al. (2009). It was identified as a crucial component of the plasma membrane repair process (Cai et al., 2009). Previous studies have demonstrated that MG53 can increase lipid accumulation in the cardiovascular system by affecting glucose uptake (Liu et al., 2015a). Furthermore, MG53 can also protect the heart against ischemia-reperfusion injury (Liu et al., 2015b). Increasing extracellular MG53 levels also plays an important role in facilitating cardiac oxidative injury (Cao et al., 2010). Other recent studies also show that serum MG53 levels are increased in patients with type 2 diabetes mellitus (Wu et al., 2019).

However, most studies on the pathophysiologic function of MG53 in cardiovascular disease have been performed in animal models. The roles and clinical significance of MG53 in humans with CAD or AMI are not well established. Therefore, in this study, we aimed to determine the clinical value of MG53 in patients with stable CAD and AMI.

## Materials and Methods

### Study Design

A total of 639 consecutive patients with suspected CAD who underwent coronary artery angiography in our facility, were included in this study. The patients comprised 222 patients with chronic stable coronary disease (CAD group), 212 patients with acute myocardial infarction (AMI group), and 205 patients without coronary artery stenosis who were included in the control group (Con group).

The CAD was defined as coronary stenosis of ≥50% in the left main coronary artery or ≥75% in at least one of the major epicardial arteries (Scanlon et al., 1999), while AMI were defined as ST segment elevation or non-ST segment elevation myocardial infarction in accordance with International guidelines (Thygesen et al., 2012). Patients with a history of events suggestive of acute myocardial infarction within 4 weeks were excluded from this study. Other exclusion criteria included severe diseases such as tumors, serious infections, autoimmune diseases, and other physical disabilities. All enrolled patients were ≥18 years old. The study was approved by the Institutional Review Board of Ruijin Hospital, Shanghai Jiao Tong University School of Medicine. Each subject provided written informed consent before enrollment. The study protocol of this study conforms to the ethical guidelines of the 1975 Declaration of Helsinki.

### MG53 Measurement

Blood samples were taken from the radial artery before angiography, and then whole blood was centrifuged at 2000 rpm for 15–20 min to acquire serum. After centrifugation, serum samples were immediately frozen at −80°C for further analysis. The concentration of MG53 was measured using Human-MG53 ELISA kits (Cat# CSB-EL024511HU) from Flarebio Biotech. All samples were tested by scientists that were blinded to the patients’ information.

### Baseline Parameters

The patients information including medical history and physical examination data were collected via face-to-face interviews. Echocardiography was performed by the same investigator during hospital admission, and the left ventricular end systolic diameter (LVESD), left ventricular end diastolic diameter (LVEDD), and left atrial diameter (LAD) were measured. The left ventricular ejection fraction (LVEF) was calculated using the Simpson method.

### Evaluation of the SYNTAX and GRACE Score

SYNTAX scores were calculated using the SYNTAX score calculator (available at www.syntaxscore.com) before the first PCI. The GRACE score was calculated using the GRACE score calculator website^1^ during hospital admission.

### Statistical Analysis

Continuous variables with normal distribution were reported as mean ± standard deviation (SD), whereas skewed distributed variables were expressed as medians ± quartiles. Categorical data were summarized as proportions with frequencies. Continuous variables were compared using one-way ANOVA test or Kruskal–Wallis tests where appropriate, while the chi-square test or Fisher’s exact test was used to analyze the differences in categorical variables. MG53 levels were log transformed or divided into tertiles for further analysis. Logistic regression analyses were used to ascertain the predictive value of MG53 level for the presence of sCAD in patients in the CAD group and those in the Con group, as well as the predictive value of MG53 level for the presence of AMI in patients with CAD and AMI. Linear regression models was used to evaluate the association between MG53 and SYNTAX scores. Statistical analyses were conducted using SPSS software (version 23.0; SPSS, Inc., Chicago, IL, United States). Statistical significance was considered as 2-tailed, with a *p*-value of <0.05.

## Results

### Baseline Characteristics

All enrolled patients were categorized into three groups. Demographic and laboratory features of the patients are reported in Table 1. Compared with the patients in the control group, subjects from the CAD and AMI groups were males, older, and had a history of alcohol use. In addition, medical history including diabetes, dyslipidemia, and chronic kidney disease (CKD) were more frequent in the CAD and AMI groups than in the control group. The values of BMI, platelet, and hemoglobin were comparable among the three groups. Indicators reflecting renal function such as creatinine, cystatin C, and eGFR were higher in the AMI groups than in the other groups. Furthermore, levels of CRP, glucose, troponin I (cTnI), and left ventricular ejection fraction (LVEF) were also worse in the AMI group, indicating a poor general condition.

**TABLE 1:** Baseline characteristics of all subjects.

	Con (*n* = 205)	CAD (*n* = 222)	AMI (*n* = 212)	*P*-value
**Demographic characteristics**				
Age (years)	62.89 ± 8.52	65.28 ± 8.43	66.35 ± 8.95	< 0.0001
Male, Sex (%)	141 (68.8)	167 (75.2)	172 (81.1)	0.014
BMI (kg/m^2^)	25.10 ± 4.70	24.62 ± 4.27	24.49 ± 3.21	0.04
Smoke (%)	85 (41.5)	96 (43.2)	105 (49.5)	0.217
Alcohol (%)	54 (26.3)	38 (17.1)	56 (26.4)	0.03
Heart Rate (beats/minute)	77 ± 11	78 ± 9	84 ± 22	0.002
Systolic pressure (mmHg)	135 ± 26	140 ± 27	120.5 ± 25	< 0.0001
Diastolic pressure (mmHg)	77 ± 15	76.5 ± 15	72.5 ± 17	0.003
**Medical history**				
Hypertension (%)	126 (61.8)	134 (60.4)	128 (60.4)	0.945
Diabetes (%)	46 (22.4)	74 (33.3)	62 (29.2)	0.043
Dyslipidemia (%)	24 (11.7)	36 (16.2)	68 (32.1)	< 0.0001
CKD (%)	13 (6.3)	24 (10.8)	43 (20.3)	< 0.0001
AF (%)	37 (18)	7 (3.2)	14 (6.6)	< 0.0001
Stroke (%)	18 (8.8)	26 (11.7)	20 (9.4)	0.255
Family history of CAD (%)	14 (6.8)	10 (4.5)	59 (27.8)	< 0.0001
**Lab. Examination**				
MG53	40.42 ± 39.82	60.21 ± 63.09	94.12 ± 48.94	< 0.0001
WBC (× 10^9^/L)	5.70 ± 2.13	6.00 ± 2.14	9.33 ± 3.60	< 0.0001
Hemoglobin (g/L)	142 ± 17	136.5 ± 20	137.5 ± 22	< 0.0001
Platelet (× 10^9^/L)	174 ± 64	190 ± 61	187 ± 64	0.001
hsCRP	0.65 ± 1.21	0.80 ± 1.4	3.00 ± 7.96	< 0.0001
Hb1Ac (%)	5.80 ± 0.60	5.90 ± 1.10	6.0 ± 1.10	0.004
NT-pro-BNP	75.5 ± 196.45	72.45 ± 133.85	421.50 ± 1784.53	< 0.0001
Fasting glucose (mmol/L)	5.45 ± 1.00	5.59 ± 1.21	6.13 ± 2.13	< 0.0001
Creatine (μmol/L)	78 ± 19	79.50 ± 24.3	81.5 ± 34	0.026
Triglyceride (mmol/L)	1.19 ± 0.8	1.25 ± 0.9	1.33 ± 0.96	0.088
Total cholesterol (mmol/L)	3.98 ± 1.2	3.86 ± 1.53	4.64 ± 1.47	< 0.0001
HDL (mmol/L)	1.13 ± 0.39	1.09 ± 0.41	1.04 ± 0.30	0.002
LDL (mmol/L)	2.38 ± 1.11	2.25 ± 1.29	2.95 ± 1.04	< 0.0001
Cystatin C	1.05 ± 0.25	1.03 ± 0.26	1.06 ± 0.42	0.121
eGFR (mL/minute/1.73m^2^)	86.70 ± 20.35	82.75 ± 21.23	80.70 ± 32.00	0.005
CK-MB (mg/L)	1.3 ± 1.0	2.2 ± 4.1	99 ± 248.3	< 0.0001
cTnI (ng/L)	0.01 ± 0	0.24 ± 0.54	18.21 ± 51.79	< 0.0001
**Echocardiography**				
LAd (mm)	39 ± 7	38 ± 5	38 ± 5	0.07
LVEDd (mm)	48 ± 6	49 ± 5	49 ± 5	0.1
LVESd (mm)	30 ± 4	30 ± 4	34 ± 5	< 0.0001
LVEF (%)	68 ± 6	67 ± 7	58 ± 10	< 0.0001

*Values are presented as mean ± standard deviation (SD), median with interquartile range, or n (%). MG53, Mitsugumin 53; AF, atrial fibrillation; BMI, body mass index; BUN, blood urea nitrogen; CKD, chronic kidney disease; eGFR, estimated glomerular filtration rate; HDL, high-density lipoprotein; hsCRP, high sensitivity C reactive protein; LAd, left atrium diameter; LDL, low-density lipoprotein; LVEDd, left ventricular end-diastolic diameter; LVEF, left ventricular ejection fraction; LVESd, left ventricular end-systolic diameter; NT-pro-BNP, pro-brain natriuretic peptide; WBC, white blood cell.*

### Associations Between Serum MG53 Levels and Other Factors

**FIGURE 1:**
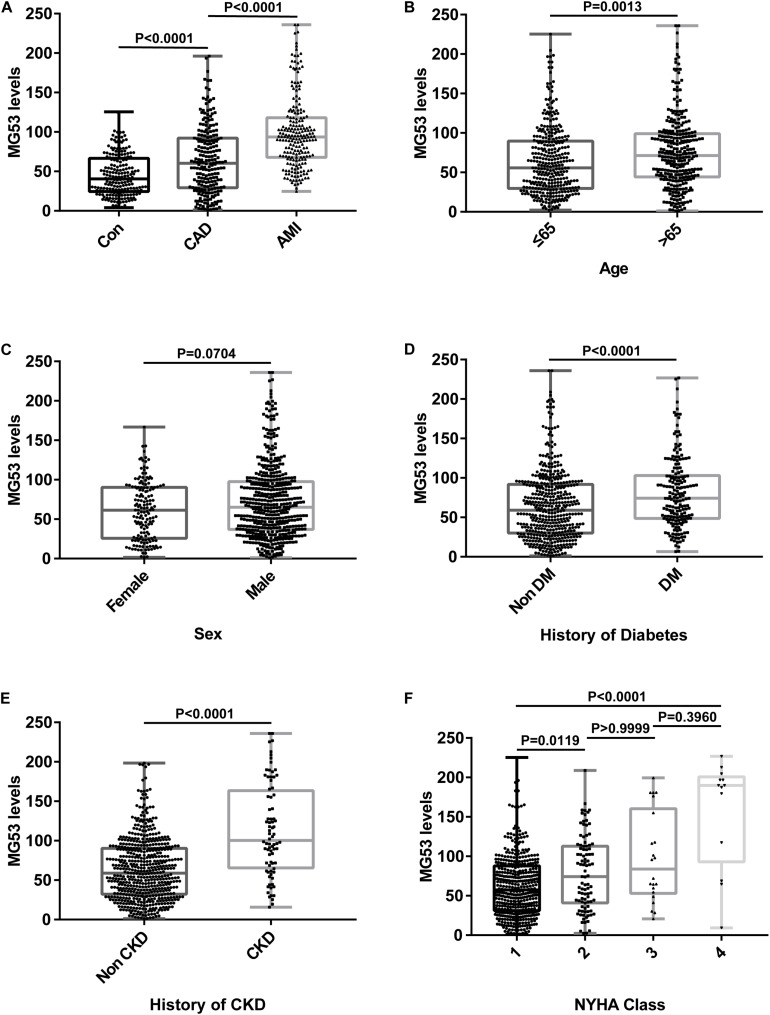
Concentration of serum MG53 in different groups. **(A)** Comparison of serum MG53 levels in patients with CAD, AMI, or negative coronary angiography. **(B)** Stratified analyses were performed by age (≤65 years/ > 65 years). **(C)** Stratified analyses were performed by gender (men/women). **(D)** Comparison of serum MG53 levels in patients with or without diabetes. **(E)** Comparison of serum MG53 levels in patients with or without CKD. **(F)** Serum mg53 levels were highest in patients with the 4th New York Heart Association (NYHA) functional class.

The serum concentration of MG53 was measured in all patients. MG53 levels were highest in patients in the AMI group. The MG53 levels in patients with stable CAD were compared to those of patients in the control group and the results are presented in Table 1 and Figure 1A. The differences of serum MG53 levels among three groups were similar to traditional markers such as CK-MB and cTnI to some extent (Supplementary Figures 1A,B). When stratified by age and gender in all subjects, the levels of MG53 were higher in patients over 65 years of age than in younger patients. However, there was no statistical significance in the serum concentration of MG53 between female and male patients (Figures 1B,C). However, MG53 levels in patients with comorbidities, such as CKD and diabetes, were significantly increased compared with those without previous such comorbidities (Figures 1D,E and Supplementary Figure 2). Furthermore, when these patients were grouped according to the New York Heart Association (NYHA) cardiac functional class, those in the fourth class showed significantly higher levels of MG53 (Figure 1F).

### MG53 Was Associated With the Prevalence of CAD and AMI

In order to clarify the diagnostic value of MG53 as a possible biomarker for stable CAD and AMI, we performed univariate and multivariate logistic regression analyses. As shown in Table 2, there was an independent correlation between log-transformed MG53 levels and increased risk for stable CAD in the CAD and control groups, when unadjusted and adjusted for age, sex, smoking, and medical history. To further demonstrate this relationship, we separated MG53 levels into three groups by tertiles. The tertiles of MG53 were also significantly related to the presence of CAD, even when adjusted for full models.

**TABLE 2:** Associations of serum MG53 with the presence of CAD.

In CAD and Control group	Unadjusted OR	*P*-value	Adjusted for Model 1 OR	*P*-value	Adjusted for Model 2 OR	*P*-value
Log MG53 per SD	1.294 (1.064–1.575)	0.01	1.291 (1.057–1.576)	0.012	1.268 (1.037–1.550)	0.021
MG53 tertiles	1.593 (1.254–2.022)	< 0.0001	1.575 (1.236–2.008)	< 0.0001	1.548 (1.214–1.972)	< 0.0001
1st	Ref		Ref		Ref	
2st	1.235 (0.774–1.971)	0.375	1.116 (0.693–1.797)	0.652	1.095 (0.679–1.767)	0.709
3st	2.551 (1.580–4.119)	< 0.0001	2.436 (1.497–3.965)	< 0.0001	2.409 (1.479–3.925)	< 0.0001

*MG53 and abnormal-distribution data were transformed into logarithmic form. The OR is shown as per 1 SD. Model 1: adjusted for age, sex, smoking, DM, dyslipidemia, hypertension, CKD Model 2: adjusted for age, sex, smoking, DM, dyslipidemia, hypertension, CKD, BMI, Hb1Ac, hsCRP, NT-pro-BNP, LVEF, LDL, creatine.*

Univariate and multivariate logistic regression models also revealed an association between serum MG53 and the prevalence of AMI in patients in the CAD and AMI groups (Table 3). These values persisted after full adjustment, both in logarithmic form or divided into tertiles.

**TABLE 3:** Associations of serum MG53 with the presence of AMI.

In CAD and AMI group	Unadjusted OR	*P*-value	Adjusted for Model 1 OR	*P*-value	Adjusted for Model 2 OR	*P*-value
Log MG53 per SD	3.319 (2.081–3.534)	< 0.0001	3.816 (2.707–5.379)	< 0.0001	2.888 (1.791–4.659)	< 0.0001
MG53 tertiles	2.323 (1.806–2.986)	< 0.0001	2.472 (1.898–3.219)	< 0.0001	2.219 (1.444–3.411)	< 0.0001
1st	Ref		Ref		Ref	
2st	3.370 (2.057–5.522)	< 0.0001	3.737 (2.241–6.232)	< 0.0001	4.570 (1.923–10.858)	0.001
3st	5.459 (3.292–9.053)	< 0.0001	6.209 (3.647–10.569)	< 0.0001	4.834 (2.013–11.612)	< 0.0001

*MG53 and abnormal-distribution data were transformed into logarithmic form. The OR is shown as per 1 SD. Model 1: adjusted for age, sex, smoking, DM, dyslipidemia, hypertension, CKD Model 2: adjusted for age, sex, smoking, DM, dyslipidemia, hypertension, CKD, BMI, Hb1Ac, hsCRP, NT-pro-BNP, LVEF, LDL, creatine, cTnI levels.*

### MG53 Was Associated With the Severity of CAD and AMI

Linear regression analysis demonstrated that log MG53 levels significantly correlated positively with SYNTAX scores in the CAD group. This result indicated that MG53 correlated with the severity of CAD. The results of multivariate linear regression further proved this relationship after full adjustment. Similar values were also observed in patients with CAD and AMI (Table 4).

**TABLE 4:** Linear regression models for SYNTAX score in different groups.

Syntax Score	Univariate analysis		Multivariable analysis		
In CAD group	β	*P*-value	β	Part. cor	*P*-value
Log MG53	0.435	< 0.0001	0.424	0.421	< 0.0001
Age	−0.088	0.193			
Gender	−0.02	0.763			
BMI	−0.055	0.416			
Creatine	0.228	0.001	0.192	0.19	0.002
hsCRP	0.115	0.089			
Hb1Ac	0.217	0.001			
LDL	0.083	0.22	0.128	0.127	0.035
DM	0.127	0.058			
Dyslipidemia	0.045	0.509			
Hypertension	0.102	0.128			
NT-pro-BNP	0.102	0.864			
LYEF	−0.045	0.507			
Smoking	−0.063	0.352			
**Syntax Score**					
**In CAD and AMI group**					
Log MG53	0.384	< 0.0001	0.349	0.345	< 0.0001
Age	0.063	0.193			
Gender	0.085	0.078			
BMI	−0.051	0.294			
Creatine	0.162	0.001			
hsCRP	0.175	< 0.0001	0.103	0.102	0.025
Hb1Ac	0.154	0.001			
LDL	0.142	0.003	0.101	0.1	0.026
DM	0.056	0.246			
Dyslipidemia	0.066	0.168			
Hypertension	0.082	0.089			
NT-pro-BNP	0.119	0.013			
LYEF	−0.186	< 0.0001			
Smoking	0.001	0.985			

*MG53 was transformed into a logarithmic form. Model: adjusted for age, sex, smoking, DM, dyslipidemia, hypertension, BMI, Hb1Ac, hsCRP, NT-pro-BNP, LVEF, LDL, creatine.*

**FIGURE 2:**
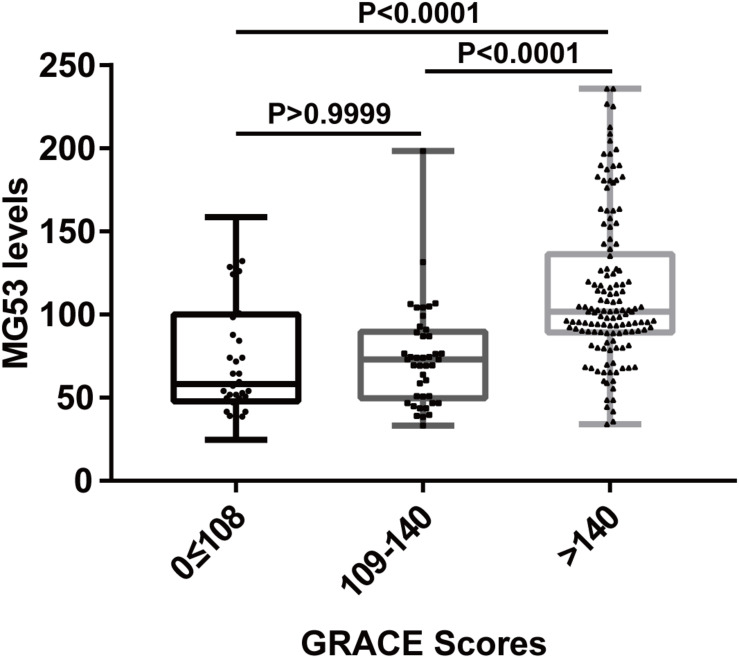
Serum MG53 levels in different groups stratified by GRACE score. The patients with AMI were divided into three groups according to their GRACE scores. Patients in the 3rd groups showed the highest MG53 level.

To verify the association between MG53 and the severity of AMI, The patients in the AMI group were divided into a high-risk group (>140), middle-risk group (109–140), and low-risk group (≤108) according to their GRACE scores. As shown in Figure 2, the serum levels of patients in the high-risk group were highest in the three groups, and the result was statistically comparable.

## Discussion

Our study has demonstrated for the first time that the serum concentration of MG53 does not only correlate with the presence of stable coronary artery disease but is also strongly associated with the prevalence of AMI in patients with CAD. Furthermore, we found that MG53 is an independent biomarker for the severity of stable CAD and AMI. We also observed that MG53 levels may be related to systemic disorders such as CKD and diabetes. To our knowledge, this is the first study on the correlation between MG53 and CAD in humans.

Coronary artery disease and its most serious complication, AMI, are major causes of morbidity and mortality worldwide (Lu et al., 2012), and both share a similar pathologic connection to atherosclerosis. While CAD is associated with lipid deposition and plaque formation, AMI is caused by atherosclerotic plaque rupture or thrombosis (Libby, 2013). The inflammatory response is an important driver of both CAD and AMI, and it is worse in the presence of factors, such as diabetes, dyslipidemia, smoking, and hypertension.

We found that serum levels of MG53 correlated with the presence of diabetes in control and CAD group. This observation is consistent with those of previous studies showing that MG53 levels could be increased in patients with obesity and type 2 diabetes (Wu et al., 2019). Wu et al. (2019), reported that in response to high serum glucose concentration, MG53 is secreted from skeletal and cardiac muscles as a secretory cytokine to regulate tissue insulin sensitivity and homeostasis. Secretory MG53 could bind to the insulin receptor and inhibit insulin signaling, while injection of exogenous MG53 can restore the insulin response. Moreover, circulating MG53 levels were also elevated in patients with CKD. Duann et al. (2015), found that MG53 is mainly expressed in the renal cortex, and downregulation of MG53 can significantly exaggerate kidney ischemic-reperfusion injury, highlighting the protective effect of MG53 in the kidneys.

The results of logistic and linear regression models demonstrated that MG53 is positively correlated with the presence and severity of stable CAD. Subjects with higher MG53 levels were more likely to have stable CAD and higher SYNTAX scores than those with low MG53 levels. In a previous study, it was reported that MG53 could aggravate lipid accumulation in the cardiovascular system (Liu et al., 2015a). Mechanistically, MG53 may impair lipid metabolism via the upregulated peroxisome proliferation-activated receptor-α (PPAR-α) signaling pathway and its downstream receptors (Liu et al., 2015a). Furthermore, genes associated with lipid metabolism are elevated in response to the overexpression of MG53 (Wang et al., 2010). These relationships could be observed in multiple animal models, suggesting that MG53 plays an important role in lipid uptake.

We also found that circulating MG53 levels were highest in patients with AMI. In addition, when the GRACE scores of AMI patients were divided into three groups, patients with higher GRACE scores had significantly elevated MG53 levels. These findings indicate that MG53 may also be related to the presence of AMI and its severity. These results are in line with those reported by Cao et al. (2010). In their study, found that MG53 was an important cardioprotective factor in cardiac ischemia-reperfusion injury. The protective effects of MG53 were attributed to the interaction with caveolin-3 (CaV3) and activation of pro-survival factors, which could facilitates ischemic preconditioning (IPC). Intracellular MG53 levels were decreased after ischemia reperfusion (I/R) injury. Injection of recombinant human-MG53 can protect hypoxic tissues against oxidative injury. More importantly, Liu et al. (2015b), reported that intravenous delivery of the recombinant MG53 could repair membrane damage to the ischemia-induced injury in porcine models. They also found that damaged myocardium could release abundant endogenous MG53 into circulation in rodent I/R models (Liu et al., 2015b). In addition, the circulating MG53 levels in mice with I/R injury correlated with the concentration of creatine kinase. More importantly, Dan et al. showed that IPC and oxidative stress could promoted MG53 secretion from perfused hearts in rodent (Shan et al., 2020). This effect was mediated by H_2_O_2_-induced PKC-δ phosphorylation at Y311. These studies may be involved in the association between elevated serum MG53 levels and AMI in humans.

Although Lemckert et al. (2016) study had reported there is very to no low level of MG53 expressed in human heart detected by western-blot. The studies of Dan et al. had proved that MG53 is present in human left ventricle tissue by using mass spectrometry analysis, and its abundance is about 1/10 of that of human skeletal muscle. They also demonstrated that MG53 could secreted from human ES cell-derived cardiomyocytes in response to low-dose H_2_O_2_ stimulation. Regardless of its low expression levels in cardiomyocytes, knockdown of MG53 with siRNA would aggravate oxidative stress injury of cardiomyocytes differentiated from human ES cells. These discrepancy between previous studies and present research might be attributed to different experimental method or differences in the antibodies used. In addition, the ages of human heart donors were various in their research. The biological functions of MG53 in human heart tissue need further fundamental research.

## Conclusion

This study reveals that serum MG53 levels were significantly associated with the presence and severity of stable CAD and AMI. Thus, MG53 could be useful, novel biomarker for diagnosing CAD and AMI, and for identifying patients at higher risk of CAD.

## Study Limitations

First, the sample size was small, and this was a single-center study. The findings of this study need to be verified in multi-center studies with a large sample size. Second, we could not evaluate the causal relationship between MG53 levels and the presence of CAD and AMI because this was a cross-sectional study. Further studies involving following-up of patients with CAD in order to assess if they develop AMI in the future are required. Third, the ages of the different groups were comparable. Hence, age-matched groups need to be used in future studies. Finally, basic research tools such as immunohistochemistry and flow cytometry need to be performed to verify the role of MG53 in the cardiovascular system in humans. Furthermore, the identifying the underlying mechanism of action of MG53 in CVD is also necessary.

## Data Availability Statement

The raw data supporting the conclusions of this article will be made available by the authors, without undue reservation.

## Ethics Statement

The studies involving human participants were reviewed and approved by Institutional Review Board of Ruijin Hospital, Shanghai Jiao Tong University School of Medicine (2018-183). The patients/participants provided their written informed consent to participate in this study.

## Author Contributions

HYX, YQW, and SF collected and analyzed the data. ZJY performed the echocardiography. TQZ, ZBZ, JWN, JN, RD, JZZ, and FHD performed PCI and collected blood samples, HH, HZ, and JXZ performed the statistical analysis. XXY and HYX designed this study and wrote the manuscript. WWQ and RYZ made critical revisions of the manuscript. All authors contributed to the article and approved the submitted version.

## Conflict of Interest

The authors declare that the research was conducted in the absence of any commercial or financial relationships that could be construed as a potential conflict of interest.

## Footnotes

1 www.outcomes-umassmed.org/grace/

## References

[B1] Agewall S. (2008). Acute and stable coronary heart disease: different risk factors. *Eur. Heart J.* 29 1927–1929. 10.1093/eurheartj/ehn321 18621774

[B2] Cai C. Masumiya H. Weisleder N. Matsuda N. Nishi M. Hwang M. (2009). MG53 nucleates assembly of cell membrane repair machinery. *Nat. Cell Biol.* 11 56–64. 10.1016/j.bpj.2008.12.1824 19043407 PMC2990407

[B3] Cao C. M. Zhang Y. Weisleder N. Ferrante C. Wang X. Lv F. (2010). MG53 constitutes a primary determinant of cardiac ischemic preconditioning. *Circulation* 121 2565–2574. 10.1161/CIRCULATIONAHA.110.954628 20516375

[B4] Doyle J. M. Gao J. Wang J. Yang M. Potts P. R. (2010). MAGE-RING protein complexes comprise a family of E3 ubiquitin ligases. *Mol. Cell* 39 963–974. 10.1016/j.molcel.2010.08.029 20864041 PMC4509788

[B5] Duann P. Li H. Lin P. Tan T. Wang Z. Chen K. (2015). MG53-mediated cell membrane repair protects against acute kidney injury. *Sci. Transl. Med.* 7:279ra236. 10.1126/scitranslmed.3010755 25787762 PMC4524523

[B6] Hatakeyama S. (2011). TRIM proteins and cancer. *Nat. Rev. Cancer* 11 792–804. 10.1038/nrc3139 21979307

[B7] Lemckert F. A. Bournazos A. Eckert D. M. Kenzler M. Hawkes J. M. Butler T. L. (2016). Lack of MG53 in human heart precludes utility as a biomarker of myocardial injury or endogenous cardioprotective factor. *Cardiovasc. Res.* 110 178–187. 10.1093/cvr/cvw017 26790476 PMC4836626

[B8] Libby P. (2013). Mechanisms of acute coronary syndromes and their implications for therapy. *N. Engl. J. Med.* 368 2004–2013. 10.1056/NEJMra1216063 23697515

[B9] Liu F. Song R. Feng Y. Guo J. Chen Y. Zhang Y. (2015a). Upregulation of MG53 induces diabetic cardiomyopathy through transcriptional activation of peroxisome proliferation-activated receptor alpha. *Circulation* 131 795–804. 10.1161/CIRCULATIONAHA.114.012285 25637627

[B10] Liu J. Zhu H. Zheng Y. Xu Z. Li L. Tan T. (2015b). Cardioprotection of recombinant human MG53 protein in a porcine model of ischemia and reperfusion injury. *J. Mol. Cell Cardiol.* 80 10–19. 10.1016/j.yjmcc.2014.12.010 25533937 PMC4512204

[B11] Lu X. Wang L. Chen S. He L. Yang X. Shi Y. (2012). Genome-wide association study in Han Chinese identifies four new susceptibility loci for coronary artery disease. *Nat. Genet.* 44 890–894. 10.1038/ng.2337 22751097 PMC3927410

[B12] Mathers C. D. Loncar D. (2006). Projections of global mortality and burden of disease from 2002 to 2030. *PLoS Med.* 3:e442. 10.1371/journal.pmed.0030442 17132052 PMC1664601

[B13] Morse E. A. (2015). Nonobstructive coronary artery disease and risk of myocardial infarction. *J. Emerg. Med.* 48:401 10.1016/j.jemermed.2015.01.027

[B14] Negre-Salvayre A. Auge N. Camare C. Bacchetti T. Ferretti G. Salvayre R. (2017). Dual signaling evoked by oxidized LDLs in vascular cells. *Free Radic. Biol. Med.* 106 118–133. 10.1016/j.freeradbiomed.2017.02.006 28189852

[B15] Newby L. K. Jesse R. L. Babb J. D. Christenson R. H. De Fer T. M. Diamond G. A. (2012). ACCF 2012 expert consensus document on practical clinical considerations in the interpretation of troponin elevations: a report of the American College of Cardiology Foundation task force on clinical expert consensus documents. *J. Am. Coll. Cardiol.* 60 2427–2463. 10.1016/j.jacc.2012.08.969 23154053

[B16] Ozato K. Shin D. M. Chang T. H. Morse H. C. III (2008). TRIM family proteins and their emerging roles in innate immunity. *Nat. Rev. Immunol.* 8 849–860. 10.1038/nri2413 18836477 PMC3433745

[B17] Rudnicki M. A. Le Grand F. McKinnell I. Kuang S. (2008). The molecular regulation of muscle stem cell function. *Cold Spring Harb. Symp. Quant. Biol.* 73 323–331. 10.1101/sqb.2008.73.064 19329572

[B18] Scanlon P. J. Faxon D. P. Audet A. M. Carabello B. Dehmer G. J. Eagle K. A. (1999). ACC/AHA guidelines for coronary angiography: executive summary and recommendations. a report of the American College of Cardiology/American Heart Association task force on practice guidelines (Committee on Coronary Angiography) developed in collaboration with the Society for Cardiac Angiography and Interventions. *Circulation* 99 2345–2357. 10.1161/01.cir.99.17.2345 10226103

[B19] Shan D. Guo S. Wu H. K. Lv F. Jin L. Zhang M. (2020). Cardiac ischemic preconditioning promotes MG53 secretion through H2O2-activated protein kinase C-delta signaling. *Circulation* 142 1077–1091. 10.1161/CIRCULATIONAHA.119.044998 32677469

[B20] Suzuki K. Komukai K. Nakata K. Kang R. Oi Y. Muto E. (2018). The usefulness and limitations of point-of-care cardiac troponin measurement in the emergency department. *Intern. Med.* 57 1673–1680. 10.2169/internalmedicine.0098-17 29434124 PMC6047987

[B21] Thygesen K. Alpert J. S. Jaffe A. S. Simoons M. L. Chaitman B. R. White H. D. (2012). Third universal definition of myocardial infarction. *Eur. Heart J.* 33 2551–2567. 10.1093/eurheartj/ehs184 22922414

[B22] Wang X. Xie W. Zhang Y. Lin P. Han L. Han P. (2010). Cardioprotection of ischemia/reperfusion injury by cholesterol-dependent MG53-mediated membrane repair. *Circ. Res.* 107 76–83. 10.1161/CIRCRESAHA.109.215822 20466981

[B23] Weisleder N. Takeshima H. Ma J. (2008). Immuno-proteomic approach to excitation–contraction coupling in skeletal and cardiac muscle: molecular insights revealed by the mitsugumins. *Cell Calcium* 43 1–8. 10.1016/j.ceca.2007.10.006 18061662 PMC3059838

[B24] Wu H. K. Zhang Y. Cao C. M. Hu X. Fang M. Yao Y. (2019). Glucose-sensitive myokine/cardiokine MG53 regulates systemic insulin response and metabolic homeostasis. *Circulation* 139 901–914. 10.1161/CIRCULATIONAHA.118.037216 30586741

