# Supplementary material for: Serum MG53/TRIM72 Is Associated With the Presence and Severity of Coronary Artery Disease and Acute Myocardial Infarction
Source: Front Physiol. 2020 Dec 17;11:617845. doi: 10.3389/fphys.2020.617845 (PMC7773634; doi:10.3389/fphys.2020.617845)

Supplement Figure 1. Concentration of serum CK-MB and cTnI in different groups

(A, B): Comparison of serum CK-MB and cTnI levels in patients with CAD, AMI, or negative coronary angiography.

Supplement Figure 2. Serum levels of MG53 in different groups stratified by the history of diabetes mellitus.

Comparison of serum MG53 levels in patients with CAD, AMI, or negative coronary angiography, which were stratified by the medical history of diabetes mellitus.

Supplement Figure 1

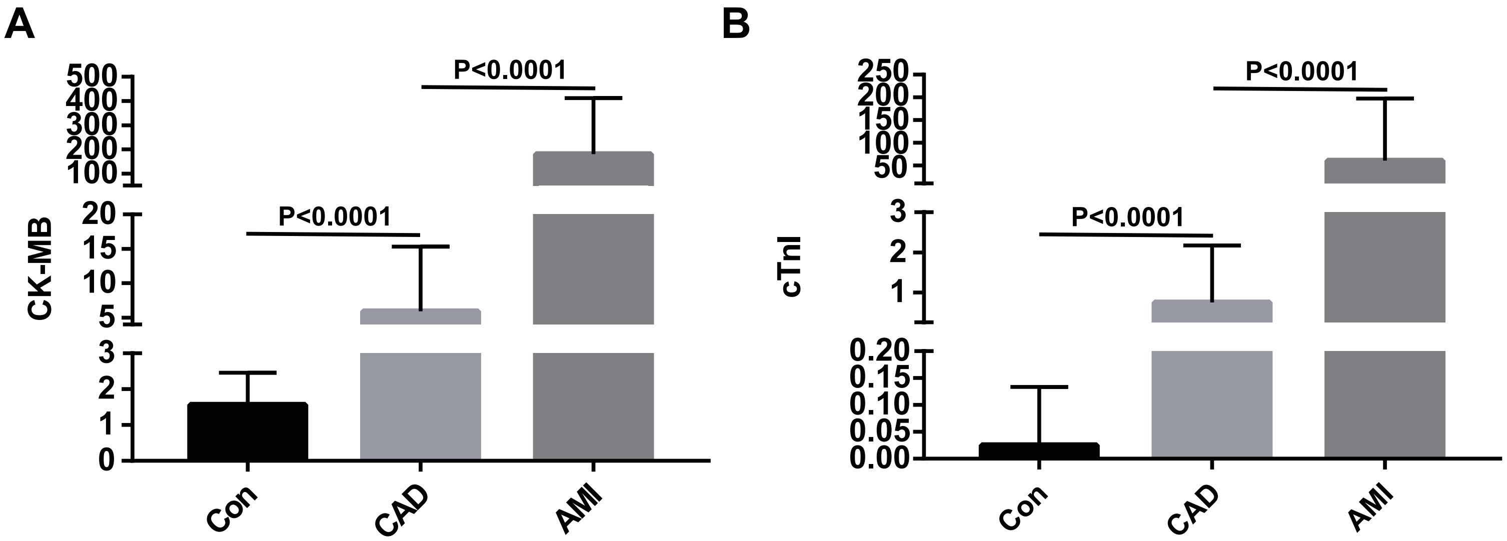

Supplement Figure 2

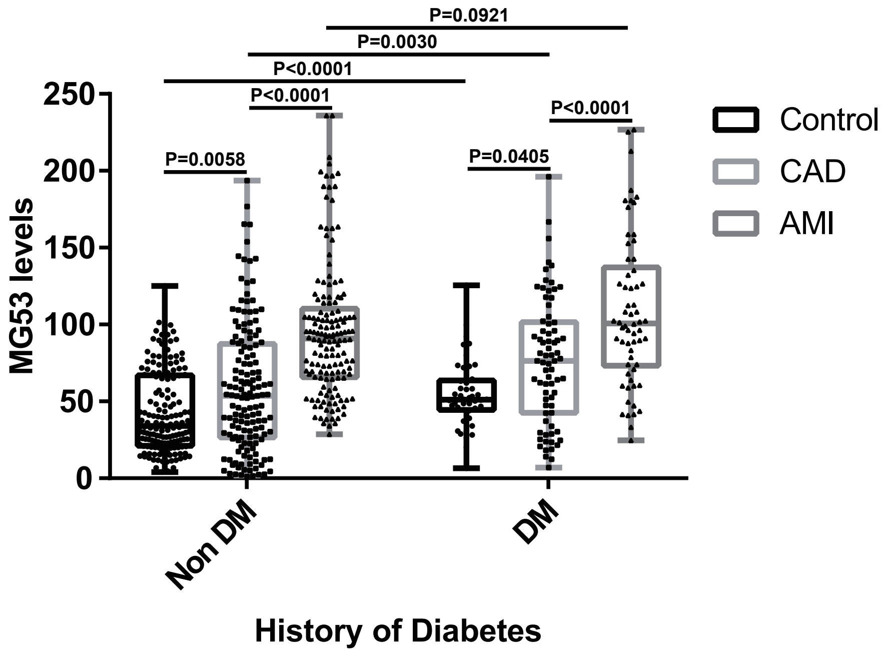

Supplement: Supplementary file 1 [file Data_Sheet_1.PDF]
